# Sequential Statistical Optimization of Media Components for the Production of Glucoamylase by Thermophilic Fungus *Humicola grisea* MTCC 352

**DOI:** 10.1155/2014/317940

**Published:** 2014-07-09

**Authors:** Vinayagam Ramesh, Vytla Ramachandra Murty

**Affiliations:** Department of Biotechnology, Manipal Institute of Technology, Manipal University, Manipal, Karnataka 576104, India

## Abstract

Glucoamylase is an industrially important enzyme which converts soluble starch into glucose. The media components for the production of glucoamylase from thermophilic fungus *Humicola grisea* MTCC 352 have been optimized. Eight media components, namely, soluble starch, yeast extract, KH_2_PO_4_, K_2_HPO_4_, NaCl, CaCl_2_, MgSO_4_ *·*7H_2_O, and Vogel's trace elements solution, were first screened for their effect on the production of glucoamylase and only four components (soluble starch, yeast extract, K_2_HPO_4_, and MgSO_4_ *·*7H_2_O) were identified as statistically significant using Plackett-Burman design. It was fitted into a first-order model (*R* ^2^ = 0.9859). Steepest ascent method was performed to identify the location of optimum. Central composite design was employed to determine the optimum values (soluble starch: 28.41 g/L, yeast extract: 9.61 g/L, K_2_HPO_4_: 2.42 g/L, and MgSO_4_ *·*7H_2_O: 1.91 g/L). The experimental activity of 12.27 U/mL obtained was close to the predicted activity of 12.15. High *R* ^2^ value (0.9397), low PRESS value (9.47), and AARD values (2.07%) indicate the accuracy of the proposed model. The glucoamylase production was found to increase from 4.57 U/mL to 12.27 U/mL, a 2.68-fold enhancement, as compared to the unoptimized medium.

## 1. Introduction

Glucoamylase or 1,4-*α*-D-glucan glucohydrolase (EC 3.2.1.3) is an industrial enzyme which can degrade amylose and amylopectin by hydrolysis of both *α*-1,4 and *α*-1,6 glucosidic links, present in starch, resulting in production of *β*-D glucose [1]. There are two stages in the production of industrial starch syrup: liquefaction and saccharification. In the first step, thermostable *α*-amylases are used to liquefy starch. Following this, saccharification is carried out at 55–60°C with fungal glucoamylases. The glucoamylase from mesophilic fungi (e.g.,* Aspergillus niger*) is unstable due to its exposure to higher temperatures for a prolonged duration [2]. This disadvantage necessitates the use of thermostable glucoamylases derived from thermophilic fungal sources [3] for industrial usage.

There are a number of thermophilic fungi such as* Thermomyces lanuginosus*,* Talaromyces duponti*,* Thermomucor indicae-seudaticae,* and* Humicola grisea* which are capable of producing glucoamylase [4–6]. Literature reveals that* Humicola grisea* is an attractive source for extracellular thermostable glucoamylase production [4, 7].* Humicola grisea* possesses efficient hydrolytic system which is responsible for the production of many polysaccharide degrading enzymes such as cellulases, amylases, trehalase, beta-glucosidase, and xylanase [8].

Glucoamylase production depends on many media components such as carbon source, nitrogen source, mineral salts, and micronutrients. Therefore it is necessary to optimize the medium components for the enhanced production of glucoamylase [5, 9–11]. The classical one-variable-at-a-time (OVAT) approach involves altering the concentration of one of the components and maintaining the others, at a specified level. This is usually problematic since it is laborious and the interaction effects between the various media components are not taken into consideration. The shortcomings of this approach are overcome by the use of statistical techniques like Plackett-Burman design (PBD), steepest ascent, and response surface methodology (RSM) [12].

Beginning from a large collection of factors, PBD helps to identify the main factors that would be taken up for further optimization processes, through lesser number of trials. The significant factors chosen from PBD are sequentially moved along the path of steepest ascent to target the maximum production of glucoamylase. The levels of the components obtained along the region of maximum response are used in central composite design (CCD), a response surface methodology technique. These form the right set of techniques leading to the optimal concentration of the various significant media components. This approach of arriving at the optimal media composition has been practiced by various researchers in many fermentation processes [13–15].

To the best of our knowledge, there are no available reports on the optimization of media components for glucoamylase production using* Humicola grisea*. Therefore, in the present report, the media components (soluble starch, yeast extract, KH_2_PO_4_, K_2_HPO_4_, NaCl, CaCl_2_, MgSO_4_ *·*7H_2_O, and Vogel's trace elements solution) for the production of glucoamylase by* Humicola grisea *MTCC 352 were optimized using response surface methodology that included a Plackett-Burman design, path of steepest ascent, and central composite design.

## 2. Materials and Methods

### 2.1. Microorganism, Inoculum Preparation, and Fermentation Conditions

The microorganism, used in the study,* Humicola grisea *MTCC 352, was obtained from Microbial Type Culture Collection, Chandigarh, India. The strain was maintained on potato dextrose agar (PDA) slant, grown at 45°C for 10 days before being stored at 4°C. The strain was subcultured, once every 2 months.

The fermentation was started with 2 mL of conidial inoculum prepared using 0.15% Triton X-100, that was added to 250 mL Erlenmeyer flasks containing 100 mL of medium (glucose 1 g, yeast extract 0.3 g, KH_2_PO_4_ 0.1 g, K_2_HPO_4_ 0.1 g, NaCl 0.1 g, CaCl_2_ 0.1 g, MgSO_4_ *·*7H_2_O 0.1 g, and 0.5 mL of Vogel's trace element solution), adjusted to pH 6. The inoculum culture was incubated at 45°C for 4 days at 150 rpm. Vogel's trace elements solution was constituted by the following, as per literature [16]: citric acid monohydrate 5 g, ZnSO_4_ *·*7H_2_O 5 g, Fe(NH_4_)_2_(SO_4_)_2_ *·*6H_2_O 1 g, CuSO_4_ *·*5H_2_O 0.25 g, MnSO_4_ *·*H_2_O 0.05 g, H_3_BO_3_ 0.05 g, and Na_2_MoO_4_ *·*2H_2_O 0.05 g, dissolved in 95 mL distilled water.

Based on preliminary experiments (data not shown), soluble starch and yeast extract showed better yields for enzyme production. Therefore, for the production medium, soluble starch was used as the carbon source in place of glucose. All the other media constituents and the culture conditions remained unaltered. Both the inoculum culture and production media were autoclaved for 15 minute at 121°C and 15 psi. The spore suspension of the fungal strain (5 mL) was inoculated in 100 mL of the production medium, taken in a 250 mL flask, for a period of 4 days.

### 2.2. Extraction of Extracellular Glucoamylase

After fermentation, the broth was subjected to filtration through Whatman No. 1 filter paper. The filtrate was centrifuged at 13000 rpm for 20 minutes to remove the fungal mycelia. The cell-free supernatant was assayed for glucoamylase activity. All the experiments were carried out in triplicate and the average values were reported.

### 2.3. Glucoamylase Activity Assay

0.05 mL of cell-free supernatant was incubated with 0.7 mL of 50 mM citrate buffer (pH 5.5) and 0.25 mL starch solution (1%, w/v) at 60°C for 10 minutes. The reaction was stopped by placing the tubes in a boiling water bath for 10 minutes. After bringing back to room temperature, the concentration of glucose formed was determined by glucose oxidase/peroxidase (GOD/POD) method [17]. One unit of glucoamylase activity was defined as the amount of enzyme that releases 1 *μ*mol glucose from soluble starch per minute under assay conditions.

### 2.4. Plackett-Burman Design

Plackett-Burman design was used to screen the media components and identify the significant components that influence the higher production of glucoamylase. Eight independent variables were chosen with three different levels, namely, low, mid, and high factor settings, coded as −1, 0, and +1, respectively, with their actual values (Table 1). These variables were screened in 13 experimental runs that included a center point, according to PBD (Table 2) along with the response (glucoamylase activity).The center point experiment was performed to obtain the standard error of the coefficients.

The Plackett-Burman design was based on the first-order model, shown in

(1) $$\begin{array}{c} G=g_{0}+\;\sum g_{i}Z_{i}, \end{array}$$

where *G* is the glucoamylase activity (U/mL), *g*_0_ is the model intercept, *g*_*i*_ is the linear coefficient, and *Z*_*i*_ is the level of the independent variable [12].

### 2.5. Path of Steepest Ascent

Following the first-order model based on PBD, new sets of experiments were performed in the direction of maximum response as described by steepest ascent method [12]. In this approach, the experiments were started at the midlevel of the statistically significant factors that were picked from PBD. The levels of the each factor were increased depending on their magnitude of the main effect. Experiments were continued until no further increase in response was observed (Table 3).

### 2.6. Central Composite Design and Response Surface Methodology

In order to obtain the optimum values of each factor, a CCD was performed. The CCD was used to obtain a quadratic model consisting of factorial points (−1, +1), star points (−2, +2), and central point (0) to estimate the variability of the process with glucoamylase yield as the response (Table 4).

Response surface methodology was employed to optimize the four selected significant factors, namely, soluble starch (*Z*_1_), yeast extract (*Z*_2_), K_2_HPO_4_ (*Z*_3_), and MgSO_4_ *·*7H_2_O (*Z*_4_), which increase the glucoamylase production. In this methodology, a 4-factor, 5-level CCD with 31 runs was employed (Table 5).

A quadratic equation was used to fit the response to the independent variables as given in (2)

(2) $$\begin{array}{c} G=g_{0}+\sum g_{i}Z_{i}+\sum g_{ii}Z_{i}^{2}+\sum gZ_{i}Z_{j}, \end{array}$$

where *G* is the predicted response of the glucoamylase activity (U/mL), *g*_0_ is the offset term (constant), *g*_*i*_ is the linear effect, *g*_*ij*_ is the quadratic effect when *i* = *j* and interaction effect when *i* < *j*, *g*_*ii*_ is the squared term, and *Z*_*i*_ and *Z*_*j*_ are the coded independent variables for statistical calculations according to

(3) $$\begin{array}{c} Z=\;\frac{R-R_{0}}{\Delta R}, \end{array}$$

where *Z* is the coded value of the independent variable, *R* is the real value of the independent variable, *R*_0_ is the real value of the independent variable on the center point, and Δ*R* is the step change value [12].

### 2.7. Statistical Analysis

Statistical analysis of the model developed by PBD and CCD was evaluated by analysis of variance (ANOVA) concept, using the statistical software package MINITAB-14 (MINITAB Inc., PA, USA). The polynomial model was statistically verified by using various parameters like linear regression coefficient *R* ^2^, *F* value, *t* value, absolute average relative deviation (AARD), and predicted residual sum of squares (PRESS).

## 3. Results and Discussion

### 3.1. Optimization by Plackett-Burman Design

The significance of the eight media components, namely, soluble starch (A), yeast extract (B), KH_2_PO_4_ (C), K_2_HPO_4_ (D), NaCl (E), CaCl_2_ (F), MgSO_4_ *·*7H_2_O (G) and Vogel's trace elements solution (H) for the production of glucoamylase was investigated as given by PBD. The response of PBD portrayed wide variation of activity from 3.02 to 6.56 U/mL (Table 6), which depicts the importance of attaining higher glucoamylase production.

Table 6 shows the main effects, coefficients, and standard error along with the *t*, *P* values and confidence levels of these components on the response (glucoamylase production). The positive and negative values of the coefficients represent the increase and decrease in glucoamylase production against the respective concentration of the components. The main effects characterize the deviations of the average between high and low levels for each one of the factors. If the main effect of a factor is positive, the glucoamylase production increases as the factor is changed from low to high level whereas the opposite behaviour (a decrease in the glucoamylase production) is observed for a negative main effect. In the current study, the media components A, B, D, E, G, and H increased the glucoamylase production at higher level whereas a decrease in response was observed for C and F components.

Based on the ANOVA results, the effects of only A (99.8%), B (99.4%), D (99%), and G (97.7%) had confidence levels greater than 95% and, hence, identified as the most significant parameters influencing glucoamylase production (Table 6).

**Figure 1:**
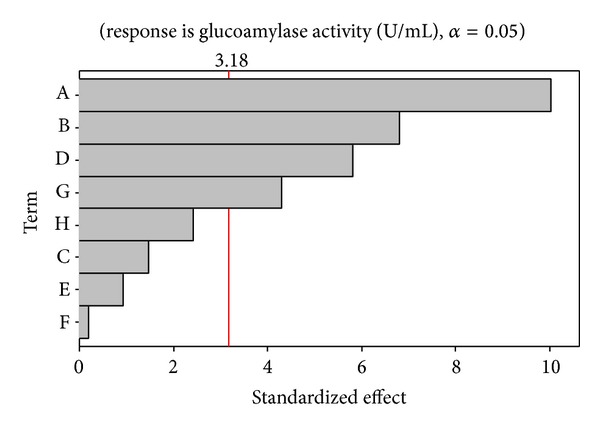
Pareto plot shows the effect of media on glucoamylase activity.

The same phenomena were explained graphically using Pareto chart (Figure 1). It explains the importance of the individual main effect of each factor to determine whether they are significantly different from zero. These values are represented by horizontal columns in the Pareto chart. For a 95% confidence level and three degrees of freedom, the *t* value equals 3.18 and is shown in the plot as a vertical line. This indicates the minimum statistically significant effect for 95% confidence level. It is clear from the Pareto chart that the four factors A, B, D, and G are significant, and therefore these four factors were taken up for further studies.

The production of glucoamylase by* Humicola grisea* depends on various types of nutrients provided. It majorly depends on type of carbon and nitrogen source used. In the present study, starch plays a significant role (positive effect) as a carbon source. Literature abounds in instances which show the prominent role played by starch as a carbon source for high glucoamylase production [5, 9, 10]. Starch (A) seems to have an “inductive effect” and portrays a significant role in glucoamylase production [11].

Similar to starch as a significant carbon source, the current study indicated the importance of yeast extract (B), as a nitrogen source, in aiding the production of glucoamylase. As in many other studies, yeast extract helps in the development of mycelial structures with a corresponding higher yield of enzymes [18]. Similar kind of results was obtained for glucoamylase production that proved the positive effect played by yeast extract [5, 6, 9].

K_2_HPO_4_ (D) was also found to have a positive effect on glucoamylase production due to the possible buffering effect on the culture medium. The fact that K_2_HPO_4_ has a positive role in the enzyme production is well in concordance with the results obtained by other researchers [5, 6, 19]. Similarly, a positive effect was observed with the addition of MgSO_4_ (G). The experimental observations are in agreement with the studies performed by Kumar and Satyanarayana [5] and Nguyen et al. [6].

Based on the regression analysis, the first-order model for the PBD with coded values is given by

(4) G=4.8542+0.0625A+0.4425B−0.0958C +0.3775D+0.0608E−0.0125F +0.2792G+0.1575H.

The *R* ^2^ value for the above model was found to be 0.9859 that implies that 98.59% of the variability of the data can be explained by the model.

From Table 2 it is clear that the glucoamylase production at the center point (4.57 U/mL) is almost close to the average glucoamylase production value (4.85 U/mL) at the factorial points which suggest that there is an absence of curvature [20, 21]. Therefore, steepest ascent method was performed to obtain the levels of the factors which were close to the optimum [22].

### 3.2. The Path Steepest Ascent

In the path of steepest ascent methodology, experiments were conducted using the four significant factors obtained from the first-order model given by PBD. This was done in order to determine the vicinity of optimum by changing the levels of the said factors with respect to the magnitude of the coefficients. By taking the center point of the four significant factors obtained from PBD (the other four factors were maintained at the low level of PBD), the path of steepest ascent was started and moved along the path in which the concentration of all the four factors increased (since all the four factors had positive effects).

The experimental design and the results of the path of steepest ascent are shown in Table 3. From the table, it is inferred that the highest response of glucoamylase activity of 11.64 U/mL was observed in Run 7, when medium composition was soluble starch 32.82 g/L, yeast extract 9.23 g/L, K_2_HPO_4_ 2.33 g/L, and MgSO_4_ *·*7H_2_O 1.98 g/L. Moreover, further increments in concentration of the media components resulted in a dip of glucoamylase production, which may be due to the inhibitory effect of high concentration of one of the components. Thus, it was obvious that the production of glucoamylase stabilized in the seventh run which proved that the media compositions were in the vicinity of optimum. Hence, this composition was chosen for further studies.

### 3.3. Central Composite Design and Response Surface Methodology

The CCD was conducted in order to determine the true optimum concentrations of the four factors (soluble starch, yeast extract, K_2_HPO_4_, and MgSO_4_ *·*7H_2_O) for glucoamylase production. The levels of the factors were chosen from the results of the path of steepest ascent (Run number 7), and the design matrix is shown in Table 5. A total of 31 experiments were performed according to the design matrix, and the experimental results are shown in Table 5 along with the predicted glucoamylase activity. The experimental results were fitted with a second-order polynomial equation as a function of the four factors with coded values and are given as follows:

(5) G=11.65−0.4863Z1+0.2604Z2+0.2946Z3 −0.1721Z4−0.2299Z12−0.5486Z22 −0.5986Z32−0.5686Z42−0.0669Z1Z2 +0.0219Z1Z3+0.2419Z1Z4+0.4456Z2Z3 +0.1531Z2Z4−0.2406Z3Z4,

where *G* is the glucoamylase activity (U/mL) and *Z*_1_, *Z*_2_, *Z*_3_, and *Z*_4_ are soluble starch, yeast extract, K_2_HPO_4,_ and MgSO_4_ *·*7H_2_O, respectively.

The ANOVA results (Table 7) obtained in the present study are in good agreement with the general facts of higher *F*, predicted *R* ^2^ values, and lower PRESS values which specify a better fit [12]. Similarly, *P* values < 0.05 indicate that the model terms were significant. In this study, all the linear, square effects of *Z*_1_, *Z*_2_, *Z*_3_, and *Z*_4_ and interactive effects of *Z*_1_ *Z*_4_, *Z*_2_ *Z*_3_, and *Z*_3_ *Z*_4_ were significant for glucoamylase production. Therefore by removing the insignificant terms (*Z*_1_ *Z*_2_, *Z*_1_ *Z*_3_, and *Z*_2_ *Z*_4_) a reduced model was obtained as follows:

(6) G=11.65−0.4863Z1+0.2604Z2+0.2946Z3 −0.1721Z4−0.2299Z12−0.5486Z22 −0.5986Z32−0.5686Z42+0.2419Z1Z4 +0.4456Z2Z3−0.2406Z3Z4.

**Figure 2:**
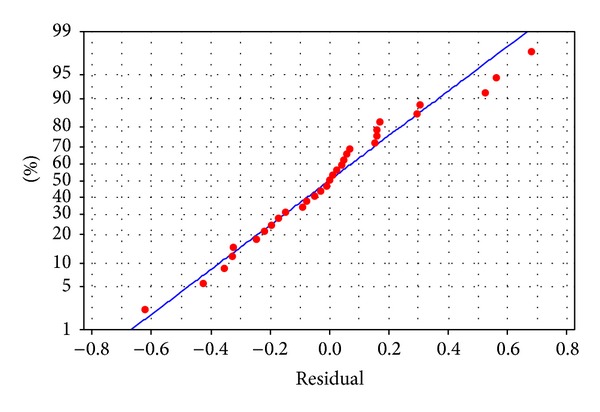
Normal probability plot of glucoamylase production.

For this reduced model, the values of various statistical parameters were as follows: *F* value: 26.89, coefficient of determination (*R* ^2^): 0.9397, predicted *R* ^2^: 0.7688, adjusted *R* ^2^: 0.9047, and PRESS: 9.47. The increase in *F* value, increase in predicted *R* ^2^ value, and a decrease in the PRESS value indicate that the reduced model fits the data in a better way. This is corroborated by the higher *F* values of the model than the *F* value of lack of fit [23, 24]. The linear trend line in Figure 2 shows that the data are normally distributed which confirms that the model fits well with the experimental results. Therefore, the major assumptions of the model (normal distribution of errors, same errors of variance, randomization, and zero mean error) stand validated.

In addition to these, another statistical parameter, AARD (%) (7), was calculated. It explains the extent to which the predicted values differ from the experimental values and a lesser value (<5%) is preferred for a good model [25]. Consider

(7) $$\begin{array}{c} \text{AARD}=\;\frac{I}{N}\overset{N}{\underset{i=1}{\sum }}\left[\frac{G_{i}^{\text{obs}}-G_{i}^{\text{pre}}}{G_{i}^{\text{obs}}}\right]\times 100, \end{array}$$

where *N* is the number of experimental data points.

For the current system, an AARD of 2.07% was obtained which implies that the model is adequate for the data.

**Figure 3:**
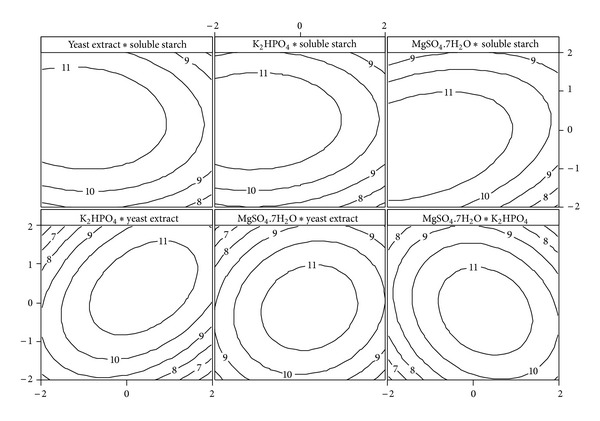
Contour plots of interaction effect of variables on glucoamylase activity (U/mL).

In order to visualize the interaction effects between each variable on glucoamylase production, two-dimensional contour plots are shown graphically in Figure 3. The interaction effects between two factors are shown with other two variables kept constant at their center value. It is clear from the plots that there is a change in glucoamylase production with respect to the low or high levels of the factors. The shape of the plot determines the extent of interaction between the factors. The elliptical shape of the contour plot between the factors, soluble starch and MgSO_4_ *·*7H_2_O and yeast extract and K_2_HPO_4,_ indicates the significant interaction effect and an increase in glucoamylase production at their higher values. The interaction effect between K_2_HPO_4_ and MgSO_4_ *·*7H_2_O indicates an elliptical shape with negative effect (decrease in glucoamylase production at higher values). The circular shape of the contour plots among the remaining variables confirms that there was no or less interaction between them.

The same phenomena are numerically shown in Table 7 (*P* < 0.05: presence of interaction and *P* > 0.05: no interaction). The reduced regression model was solved for maximum glucoamylase production using the response optimizer tool in MINITAB 14.0 and the optimum levels of each variable in uncoded units were as follows: soluble starch = 28.41 g/L, yeast extract = 9.61 g/L, K_2_HPO_4_ = 2.42 g/L, and MgSO_4_ *·*7H_2_O = 1.91 g/L, all of which were located within the experimental range. The predicted glucoamylase activity under these optimum conditions was 12.15 U/mL.

### 3.4. Experimental Verification of the Model

In order to validate these results, experiments were done in triplicate at the optimized values. Under the optimized conditions, the predicted response for glucoamylase activity was 12.15 U/mL and the average of observed experimental values was 12.27 ± 0.16 U/mL. The good correlation between the observed and predicted values confirms the adequacy of the model. This optimization strategy led to the enhancement of glucoamylase production from 4.57 U/mL (unoptimized medium) to 12.27 U/mL (optimized medium), a 2.68-fold increase. In addition to this, the optimized glucoamylase activity was found to be higher than the available literature value for various thermophilic fungi such as* Scytalidium thermophilum* 15.8 (3.62 U/mL) [26],* Thermomyces lanuginosus* A.13.37 (2.8 U/mL) [2], and* Thermomyces lanuginosus* ATCC 200065 (7.4 U/mL) [27] and comparable with* Thermomyces lanuginosus* TO3 (13 U/mL) [28].

## 4. Conclusion

In the present study, we have demonstrated the use of statistical design for the rapid identification and optimization of significant media components for the production of glucoamylase by thermophilic fungus* Humicola grisea* MTCC 352 which resulted in 2.68-fold enhancement of glucoamylase activity as compared to the unoptimized medium. Initially, eight media components were screened using PBD for their effect on the production of glucoamylase. Out of them, soluble starch, yeast extract, K_2_HPO_4_, and MgSO_4_ *·*7H_2_O were found to be statistically significant. The method of steepest ascent identified the region of optimum. The values of the four parameters were optimized by employing CCD (soluble starch: 28.41 g/L, yeast extract: 9.61 g/L, K_2_HPO_4_: 2.42 g/L, and MgSO_4_ *·*7H_2_O: 1.91 g/L). The proposed second-order model was validated as the difference between the obtained experimental activity of 12.27 U/mL and the predicted activity of 12.15 was meagre. Thus, the optimized media composition found out in the present investigation might reduce the overall cost of the medium and provides a basis for further studies on the large scale glucoamylase production.

## Figures and Tables

**Table 1:** Different levels of experimental variables used for the production of glucoamylase using Plackett-Burman design.

Symbol	Variable	Units	Coded level		
			−1	0	1
*A*	Soluble starch	(g/L)	5	10	15
*B*	Yeast extract	(g/L)	1	3	5
*C*	KH_2_PO_4_	(g/L)	0.5	1	1.5
*D*	K_2_HPO_4_	(g/L)	0.5	1	1.5
*E*	NaCl	(g/L)	0.5	1	1.5
*F*	CaCl_2_	(g/L)	0.5	1	1.5
*G*	MgSO_4_ *·*7H_2_O	(g/L)	0.5	1	1.5
*H*	Vogel's solution	(mL)	0.1	0.5	1

**Table 2:** Plackett-Burman experimental design matrix for glucoamylase production.

Run	*A*	*B*	*C*	*D*	*E*	*F*	*G*	*H*	Glucoamylase activity (U/mL)
1	1	1	−1	1	−1	−1	−1	1	6.31
2	−1	1	1	1	−1	1	1	−1	4.87
3	−1	1	1	−1	1	−1	−1	−1	3.78
4	−1	−1	−1	−1	−1	−1	−1	−1	3.02
5	−1	−1	−1	1	1	1	−1	1	3.97
6	1	−1	1	1	−1	1	−1	−1	4.94
7	−1	1	−1	−1	−1	1	1	1	4.83
8	0	0	0	0	0	0	0	0	4.57
9	1	1	1	−1	1	1	−1	1	5.43
10	−1	−1	1	1	1	−1	1	1	4.74
11	1	−1	1	−1	−1	−1	1	1	4.79
12	1	1	−1	1	1	−1	1	−1	6.56
13	1	−1	−1	−1	1	1	1	−1	5.01

**Table 3:** Experimental results of the path of steepest ascent.

Variable	Soluble starch (g/L)	Yeast extract (g/L)	K_2_HPO_4_ (g/L)	MgSO_4_ *·*7H_2_O (g/L)	Glucoamylase activity (U/mL)
Base point (zero level in the PBD)	10	3	1	1	
Origin step unit (concentration range of unity level)	5	2	0.5	0.5	
Slope (estimated coefficient ratio from equation)	0.6525	0.4425	0.3775	0.2792	
New step unit (slope × origin step unit)	3.26	0.89	0.19	0.14	
Run 1	13.26	3.89	1.19	1.14	5.16
Run 2	16.52	4.78	1.38	1.28	5.91
Run 3	19.78	5.67	1.57	1.42	6.88
Run 4	23.04	6.56	1.76	1.56	8.13
Run 5	26.3	7.45	1.95	1.7	9.42
Run 6	29.56	8.34	2.14	1.84	10.48
Run 7	32.82	9.23	2.33	1.98	11.64
Run 8	36.08	10.12	2.52	2.12	10.28
Run 9	39.34	11.01	2.71	2.26	9.67
Run 10	42.6	11.9	2.9	2.4	9.21

**Table 4:** Ranges of the independent variables used in central composite design.

Symbol	Variable	Unit	Coded level				
			−2	−1	0	1	2
*Z*_1_	Soluble starch	(g/L)	26.3	29.56	32.82	36.08	39.34
*Z*_2_	Yeast extract	(g/L)	7.45	8.34	9.23	10.12	11.01
*Z*_3_	K_2_HPO_4_	(g/L)	1.95	2.14	2.33	2.52	2.71
*Z*_4_	MgSO_4_ *·*7H_2_O	(g/L)	1.7	1.84	1.98	2.12	2.26

**Table 5:** Central composite design matrix for the experimental design and predicted responses for glucoamylase activity.

Trial	Coded variable level				Glucoamylase activity (U/mL)	
	*Z*_1_	*Z*_2_	*Z*_3_	*Z*_4_	Observed	Predicted
1	−1	−1	1	−1	10.73	10.498
2	0	0	0	0	11.56	11.65
3	1	1	1	1	10.2	10.156
4	0	0	0	0	11.72	11.65
5	0	0	2	0	9.42	9.845
6	0	0	0	2	8.41	9.031
7	1	1	−1	−1	8.38	8.186
8	−1	−1	−1	−1	10.56	10.362
9	−1	−1	1	1	8.93	8.883
10	−1	1	1	−1	11.87	11.737
11	−1	1	−1	1	10.22	9.779
12	1	1	1	−1	10.4	10.191
13	−2	0	0	0	11.35	11.703
14	0	0	0	0	11.82	11.65
15	1	−1	−1	1	9.42	9.311
16	1	−1	−1	−1	8.96	8.996
17	2	0	0	0	9.54	9.758
18	0	0	0	0	11.62	11.65
19	0	0	0	−2	9.77	9.72
20	0	0	0	0	11.48	11.65
21	1	−1	1	−1	9.02	9.219
22	−1	1	−1	−1	9.56	9.82
23	0	0	−2	0	8.52	8.666
24	0	−2	0	0	8.69	8.935
25	0	0	0	0	11.71	11.65
26	−1	1	1	1	11.1	10.735
27	0	0	0	0	11.64	11.65
28	1	1	−1	1	9.21	9.113
29	1	−1	1	1	9.16	8.571
30	0	2	0	0	9.65	9.976
31	−1	−1	−1	1	9.83	9.71

**Table 6:** Statistical analysis of PBD on glucoamylase activity.

Variables	Main effect	Coefficients	Standard error	*t* value	*P* value	Confidence level (%)
Intercept		4.8542	0.065	74.68	<0.0001	
*A*	1.3050	0.6525	0.065	10.04	0.002	99.80∗
*B*	0.8850	0.4425	0.065	6.81	0.006	99.40∗
*C*	−0.1917	−0.0958	0.065	−1.47	0.237	76.30
*D*	0.7550	0.3775	0.065	5.81	0.010	99.00∗
*E*	0.1217	0.0608	0.065	0.94	0.418	58.20
*F*	−0.0250	−0.0125	0.065	−0.19	0.860	14.00
*G*	0.5583	0.2792	0.065	4.29	0.023	97.70∗
*H*	0.3150	0.1575	0.065	2.42	0.094	90.60

*Statistically significant at 95% confidence level.

**Table 7:** Analysis of variance (ANOVA) for the fitted second-order polynomial model for optimization of glucoamylase production.

Variables	Coefficient estimate	Sum of squares	Degrees of freedom	*F* value	*P* value
Model	11.65	38.9579	14	22.06	<0.0001
*Z*_1_	−0.48625	10.0955	1	44.98	<0.0001
*Z*_2_	0.260417	5.6745	1	12.9	0.002
*Z*_3_	0.294583	1.6276	1	16.51	0.001
*Z*_4_	−0.172083	2.0827	1	5.63	0.03
*Z*_1_ *Z*_1_	−0.229896	0.1854	1	11.98	0.003
*Z*_2_ *Z*_2_	−0.548646	5.5598	1	68.23	<0.0001
*Z*_3_ *Z*_3_	−0.598646	8.3763	1	81.23	<0.0001
*Z*_4_ *Z*_4_	−0.568646	9.2467	1	73.3	<0.0001
*Z*_1_ *Z*_2_	−0.066875	0.0716	1	0.57	0.462
*Z*_1_ *Z*_3_	0.021875	0.0077	1	0.06	0.809
*Z*_1_ *Z*_4_	0.241875	0.9361	1	7.42	0.015
*Z*_2_ *Z*_3_	0.445625	3.1773	1	25.19	<0.0001
*Z*_2_ *Z*_4_	0.153125	0.3752	1	2.97	0.104
*Z*_3_ *Z*_4_	−0.240625	0.9264	1	7.34	0.015
Residual		2.0185	16		
Lack of fit		1.9431	10	15.46	0.002
Pure error		0.0754	6		

Total		40.9763	30		

*R* ^2^ = 95.07%, *R* ^2^_Adj_ = 90.76%, *R* ^2^_Pre_ = 72.44%, and PRESS: 11.29.
